# FreqPose: Frequency-Aware Diffusion with Fractional Gabor Filters and Global Pose–Semantic Alignment

**DOI:** 10.3390/s26041334

**Published:** 2026-02-19

**Authors:** Meng Wang, Bing Wang, Huiling Chen, Jing Ren, Xueping Tang

**Affiliations:** 1 Faculty of Information Engineering and Automation, Kunming University of Science and Technology, 727 South Jingming Road, Kunming 650500, China; 20232204231@stu.kust.edu.cn (B.W.);; 2 Faculty of Electrical Engineering, Kunming University of Science and Technology, 727 South Jingming Road, Kunming 650500, China

**Keywords:** pose-guided person image synthesis, diffusion models, fractional gabor filters, frequency-aware feature extraction, global semantic-pose alignment, high-fidelity texture reconstruction

## Abstract

The task of pose-guided person image generation has long been confronted with two major challenges: high-frequency texture details tend to blur and be lost during appearance transfer, while the semantic identity of the person is difficult to maintain consistently during pose changes. To address these issues, this paper proposes a diffusion-based generative framework that integrates frequency awareness and global semantic alignment. The framework consists of two core modules: a multi-level fractional-order Gabor frequency-aware network, which accurately extracts and reconstructs high-frequency texture features such as hair strands and fabric wrinkles, enhances image detail fidelity through fractional-order filtering and complex domain modeling; and a global semantic-pose alignment module that utilizes a cross-modal attention mechanism to establish a global mapping between pose features and appearance semantics, ensuring pose-driven semantic alignment and appearance consistency. The collaborative function of these two modules ensures that the generated results maintain structural integrity and natural textures even under complex pose variations and large-angle rotations. The experimental results on the DeepFashion and Market1501 datasets demonstrate that the proposed method outperforms existing state-of-the-art approaches in terms of SSIM, FID, and perceptual quality, validating the effectiveness of the model in enhancing texture fidelity and semantic consistency.

## 1. Introduction

**Figure 1:**
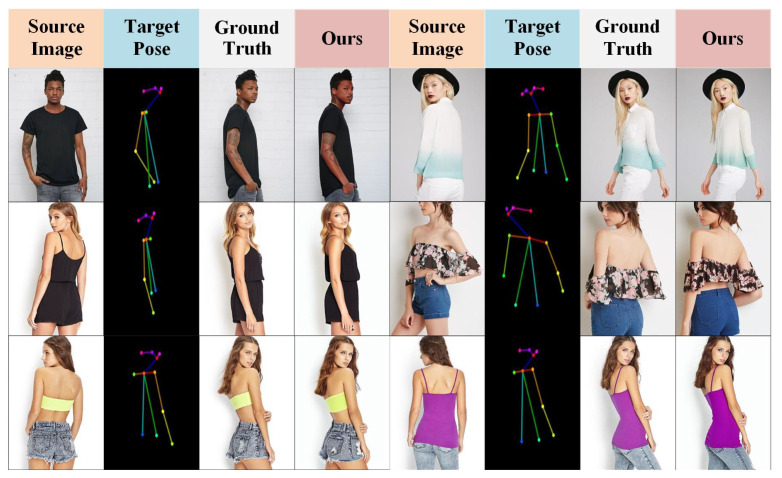
Illustration of pose-guided person image synthesis.

Pose-guided person image generation (PGPIS) aims to generate person images with a target pose while maintaining consistent identity information (as shown in Figure 1). This task requires that the generated images not only preserve identity consistency and semantic coherence, but also exhibit realistic texture details and high visual quality, matching the target pose. PGPIS has significant applications in various fields. For instance, in animation production, it can automatically generate high-resolution character images in different poses, saving time spent manually drawing. It also plays an important role in virtual try-on, game character customization, and special effects production, where it helps increase automation, reduce production costs, and provide users with an enhanced visual experience. In PGPIS, accurately transferring high-frequency texture details and maintaining stable person-specific semantic information are the key challenges for achieving high-fidelity generation. The former requires the reproduction of details such as fabric textures, hair strands, and skin texture, while the latter necessitates precise pose alignment and the avoidance of semantic distortion. To address these challenges, current research primarily follows two technological paths: Generative Adversarial Networks (GANs) [1] and diffusion models [2].

GAN-based approaches pioneered the PGPIS paradigm and explored how to balance appearance detail and pose control. XingGAN [3] processes appearance and pose in dual branches and fuses them via collaborative attention, but its complex structure and imperfect fusion lead to remaining visual artifacts. MARLM-GAN [4] improves global relationships through inter-activation and residual linear modeling, yet struggles with large pose changes and occlusions due to the limitations of single-image information. RSA-GAN [5] incorporates human parsing priors to enhance realism but incurs high training costs and performs poorly in low-resolution or complex scenes. UPG-GAN [6] combines CycleGAN and VAE to enable unpaired training and diverse generation, though it suffers from low resolution and weak handling of rare poses and facial areas. DPIG [7] disentangles the foreground, background, and pose in a two-stage framework, but insufficient structural consistency and the absence of skip connections limit detail reconstruction. Despite their foundational role, GANs face inherent issues—including instability, mode collapse, limited diversity, and difficulty modeling complex semantics and high-frequency textures—making them less suitable for modern high-resolution PGPIS tasks. Diffusion models alleviate these issues through stable Markov-based optimization, avoiding mode collapse and supporting rich semantic conditioning, yet still struggle with fine-grained texture control, component-level semantics, and occlusion handling. Although recent diffusion-based improvements have targeted PGPIS, they continue to expose new limitations.

PIDM [8] encodes multi-scale textures and injects them into UNet cross-attention to link source appearance with target pose, but it lacks explicit body part modeling and struggles with high-frequency detail under large pose changes. IMAGPose [9] combines the VAE and CLIP encoders for texture and semantic extraction, yet VAE compression causes detail loss and CLIP lacks component-level understanding, leading to incorrect texture mappings. CFLD [10] decouples multi-scale features with Swin Transformer and refines them via attention, but still fails to model complex garment components, often causing texture misalignment. PoCoLD [11] performs latent diffusion guided by DensePose, but VAE downsampling discards details and lacks multi-view consistency. HumanSD [12] conditions diffusion on text and pose but does not extract appearance features, making fine textures difficult to preserve. RePoseDM [13] attempts texture–pose alignment via attention, yet feature correspondence remains weak, leading to misalignment and distortion under large pose variations. DNAF [14] injects Swin-extracted style features as constant conditions and uses noise-aware features for detail enhancement, but may cause perceptual distortion. UPGPT [15] extracts region-specific features via CLIP, but the pipeline is complex and CLIP’s semantic bias harms fine-grained texture fidelity. TCAN [16] uses an appearance UNet pre-trained on Realistic Vision to improve consistency, though the system is heavy, costly, and reliant on multiple cooperating modules.

**Figure 2:**
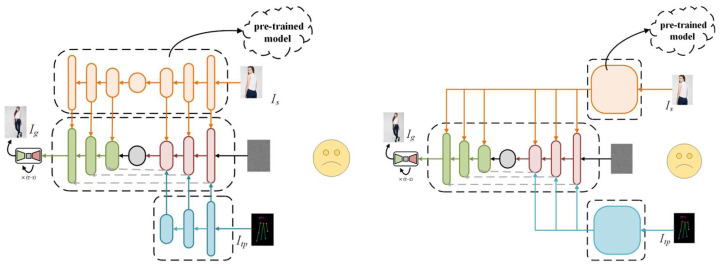
Analysis of limitations in existing source image processing. On the **right**, direct encoding methods (e.g., VAE) are shown, while the **left** illustrates condition injection methods (e.g., Swin Transformer). Current approaches suffer from a lack of frequency modeling and semantic alignment mechanisms, resulting in semantic misalignment and blurred high-frequency details. The “sad face” icons denote the specific loss of texture fidelity in these methods.

In summary, existing source image processing methods mainly fall into two categories (as shown in Figure 2): direct and multi-level encoding in the real domain, and direct embedding strategies lacking semantic alignment mechanisms. However, both categories struggle with preserving fine textures and achieving component-level semantic understanding. Most rely on compressed pre-trained encoders (e.g., VAE, CLIP, Swin Transformer, and ControlNet) that model appearance only in the real domain while ignoring magnitude–phase information, leading to high-frequency detail loss. Consequently, although global structures can be produced, facial blurriness and texture distortion remain common. Even multi-scale or noise-enhanced methods suffer from texture misalignment and regional detail loss. Moreover, current approaches lack precise body part understanding and fail to achieve interactive alignment between pose and appearance features, resulting in inconsistent mapping between the source’s appearance and the target pose.

Despite providing a powerful generative prior for person image generation, diffusion models still face the dual challenge of detail preservation and semantic alignment when processing source images. Achieving high-fidelity texture reconstruction and precise component-level semantic control without sacrificing generation efficiency remains a key direction for future research.

To overcome the limitations of general-purpose pre-trained encoders in texture detail preservation and component-level semantic understanding due to their modeling of texture details only in the real domain, this work proposes a texture feature extraction network based on fractional-order Gabor frequency awareness (MLGFN). This approach abandons traditional real-domain modeling and adopts a multi-level complex frequency-aware architecture to capture texture details from multiple perspectives. By utilizing fractional-order Gabor filters, both the amplitude and phase information of textures are captured simultaneously. The combination of frequency-aware units and frequency-domain self-attention mechanisms enables the more precise modeling of texture directional features and long-range dependencies. Compared to traditional methods, MLGFN significantly improves the retention of high-frequency textures, enhances the fidelity of facial features and fabric details, and provides more accurate component-level semantic understanding. To address issues of semantic distortion and pose misalignment, we also introduce a Global Semantic–Pose Alignment Module (GSPAM). Through a cross-attention mechanism, this module enables adaptive matching between the pose and the global semantic features, effectively improving structural consistency and texture fidelity under pose variations and in complex clothing scenarios. The source code is available at: https://github.com/bing32475/FrGPose, accessed on 15 February 2026. The main contributions of this paper are as follows:

- We proposed a texture feature extraction network based on fractional-order Gabor frequency-awareness, enhancing the capture of both amplitude and phase features of textures. This reduces high-frequency detail loss from aggressive downsampling in traditional encoders, improving the fidelity of fine-grained structures such as fabric, hair, and facial textures.
- We design a global semantic–pose alignment mechanism based on cross-attention, which combines global appearance semantics from the Swin Transformer with deep pose features. By aligning the semantic pose in the global feature domain for image reconstruction, this method ensures structural consistency and texture continuity in scenarios with large pose variations and complex garments.
- An end-to-end multi-scale feature fusion generation framework is proposed, combining frequency-aware texture networks with global pose–appearance alignment. This unified framework optimizes texture detail preservation and semantic structure modeling, achieving superior visual quality and semantic consistency across multiple datasets.

## 2. Related Work

### 2.1. Texture Preservation Approaches for PGPIS

In PGPIS, preserving fine textures under pose changes remains a significant challenge, and recent progress highlights the importance of frequency-domain cues for improving detail fidelity. Early GAN-based methods adopted encoder–decoder architectures but suffered from training instability and poor texture accuracy. Diffusion-based approaches have improved robustness, yet they remain insufficient in generating high-frequency details. These limitations indicate that relying solely on image–space features is inadequate and that frequency-domain signals are essential for high-fidelity texture recovery. However, existing frequency-domain methods lack effective directional feature extraction and amplitude–phase joint modeling. The Res FFT-Conv Block [17] improves global consistency but utilizes isotropic FFT features lacking directional selectivity, while Yang et al. [18] separate amplitude and phase using FFC but lack complex-domain modeling, thereby limiting control over structured textures.

### 2.2. Global Semantic–Pose Alignment Mechanism

Accurate semantic and pose alignment remains a fundamental challenge in person image generation. Existing appearance flow methods achieve alignment by predicting coordinate offsets; however, they are susceptible to flow distortion under large pose variations, leading to structural and textural deformation. While attention-based alignment methods improve semantic correspondence, they often result in semantic ambiguity in detailed regions due to a lack of fine-grained spatial constraints or difficulties in modeling complex spatial relations. Furthermore, although recent studies attempt to decouple style and texture, or introduce strong supervision signals, they are constrained by the absence of explicit structural priors and detail degradation during latent diffusion, making it difficult to ensure alignment accuracy and global consistency under large-scale or non-rigid transformations.

To address these challenges, we propose a unified framework comprising a fractional-order Gabor frequency-aware network and a GSPAM module. The Gabor network leverages fractional filters and complex-domain attention to capture directionally selective texture primitives, ensuring high-fidelity structural preservation. Concurrently, GSPAM aligns global appearance with pose features via cross-attention and multi-level fusion. This synergy establishes fine-grained correspondences, effectively minimizing semantic fragmentation and pose drift in complex regions such as joints and clothing boundaries.

## 3. The Proposed Framework

### 3.1. Preliminaries

#### 3.1.1. Stable Diffusion

We first review the foundations that our framework builds upon, including Stable Diffusion and two classic time-frequency transforms. Stable Diffusion (Rombach et al., 2022) [2] is an efficient diffusion probabilistic model that operates in the latent space to reduce computational costs, enabling high-resolution image synthesis. Its core lies in the noise addition and denoising processes. In this work, we extend its conditional injection mechanism by introducing dual guidance with semantic–pose alignment features and appearance detail encoding. Specifically, the noise addition process follows forward diffusion, where Gaussian noise is gradually added in the latent space $z_{0}$. For a time step t∼U[1,T], the noise addition formula is

(1) $$z_{t}=\sqrt{{\bar{\alpha }}_{t}}z_{0}+\sqrt{1-{\bar{\alpha }}_{t}}\varepsilon_{t},\;\varepsilon_{t}∼N\left(0,I\right)$$

where $\bar{\alpha_{t}}=\prod_{s=1}^{t}\left(1-\beta_{s}\right)$ is the cumulative variance schedule coefficient, and $\beta_{s}$ represents the noise schedule (either linear or cosine schedule). The denoising process reverses the diffusion via ${\mathrm{UNet}}_{\theta }$, predicting the added noise:

(2) $$L=E_{t,z_{0},\epsilon }\left[{∥\epsilon -\epsilon_{\theta }\left(z_{t},t,c\right)∥}^{2}\right]$$

Here, *c* represents the conditional input, which is injected into the UNet through cross-attention. The iterative denoising process begins from pure noise $z_{T}∼N\left(0,I\right)$ and progressively restores $z_{0}$, which is then decoded into the image $\hat{X}=D\left(z_{0}\right)$ by the VAE decoder *D*. This core formula ensures the stability of the generation process. Based on this framework, we fine-tune the model to integrate semantic–pose alignment and appearance detail perception features, achieving the simultaneous optimization of texture fidelity and pose consistency.

#### 3.1.2. Gabor Transform and Fractional Fourier Transform

The 2D Gabor transform is a widely used time–frequency analysis method that performs localized frequency decomposition by multiplying a Gaussian window with a complex exponential wave. Unlike the global Fourier transform, it preserves local spatial–frequency information, enabling effective analysis of regional signal variations. For a 2D signal f(x,y), the Gabor transform is defined as

(3) $$\begin{array}{cc} G(x_{0},y_{0},u_{0},v_{0}) & =\;\int \int \;f\left(x,y\right)\;g^{*}\left(x-x_{0},y-y_{0}\right)\;\\ \; & \;\times \;e^{-j2\pi (u_{0}x+v_{0}y)}\;dx\;dy \end{array}$$

where ∗ denotes the complex conjugate operator. The window function g(x,y) is typically chosen as a Gaussian function. The Gaussian function localizes the signal in the spatial domain, while the complex exponential term performs frequency-domain analysis. The window function is expressed as

(4) $$g\left(x,y\right)=\exp\left(-\frac{x^{2}+\gamma^{2}y^{2}}{2\sigma^{2}}\right)$$

where γ is the aspect ratio controlling the scale difference between the two spatial directions, and σ determines the width of the window function. To construct the Gabor filter, this real-valued envelope is modulated by a complex sinusoidal carrier. The specific 2D Gabor filter kernel is formulated as

(5) $$g\left(x,y\right)=\exp\left(-\frac{x_{\theta }^{2}+\gamma^{2}y_{\theta }^{2}}{2\sigma^{2}}\right)e^{j\left(\frac{2\pi x_{\theta }}{\lambda }+\phi \right)}$$

where λ represents the wavelength of the sinusoidal factor, θ denotes the orientation of the parallel stripes, and ψ is the phase offset. The rotated coordinates $\left(x_{\theta },y_{\theta }\right)$ are defined as $x_{\theta }=x\cos\theta +y\sin\theta$ and $y_{\theta }=-x\sin\theta +y\cos\theta$. This transform allows local frequency analysis in multiple orientations and is widely applied in image edge detection, texture analysis, and other computer vision tasks.

The traditional Fourier transform (FT) can be viewed as a 90° rotation of the signal in the time–frequency plane, while the fractional Fourier transform (FRFT) introduces a more flexible joint time–frequency analysis framework by controlling arbitrary rotations through the fractional order parameter α, making it suitable for the analysis of local and non-stationary signals.

The FRFT is mathematically defined as

(6) $$X_{\alpha }\left(u\right)=\int_{-\infty }^{+\infty }x\left(t\right)K_{\alpha }\left(t,u\right)dt$$

where the kernel function $K_{\alpha }\left(t,u\right)$ is given by

(7) $$K_{\alpha }\left(t,u\right)=\sqrt{\frac{1-j\cot\alpha }{2\pi }}\exp\left[j\frac{t^{2}+u^{2}}{2}\cot\alpha -jtu\csc\alpha \right]$$

Here, α represents the fractional order that determines the rotation angle in the time–frequency plane. When α=π/2, the FRFT reduces to the conventional Fourier transform, and when α=0, it corresponds to the original signal.

In the two-dimensional case, FRFT performs analysis by rotating the time–frequency plane along both axes. The 2D FRFT is defined as

(8) $$F_{\alpha_{x},\alpha_{y}}\left(u,v\right)=\;\int \int \;f\left(x,y\right)K_{\alpha_{x}}\left(x,u\right)K_{\alpha_{y}}\left(y,v\right)dxdy$$

where $K_{\alpha_{x}}\left(x,u\right)$ and $K_{\alpha_{y}}\left(y,v\right)$ are the FRFT kernels along the x- and y-directions, respectively. This formulation enables representation of 2D signal characteristics at arbitrary rotation angles in the time–frequency plane.

#### 3.1.3. Fractional Gabor Transform

The fractional Gabor transform (FGT) integrates the FRFT with the conventional Gabor transform, enabling local frequency analysis in arbitrary orientations through a rotated time–frequency plane. By applying Gabor filtering within the FRFT domain, FGT offers more flexible and precise local representations, making it well suited for directional or non-stationary signal analysis.

The general definition of the 2D fractional Gabor transform is as follows:

(9) $$G_{\alpha }\left(u,\xi \right)=\;\int \int \;f\left(r\right)g_{\alpha }^{*}\left(r-u\right)K_{\alpha_{x}}\left(x,\xi \right)K_{\alpha_{y}}\left(y,\eta \right)dxdy$$

where f(x,y) is the input signal, $g_{\alpha }\left(x,y\right)$ is the fractional Gabor window function, $K_{\alpha_{x}}\left(x,\xi \right)$ and $K_{\alpha_{y}}\left(y,\eta \right)$ are the FRFT kernels in the x- and y-directions, $u=(u_{x},u_{y})$ denotes the spatial displacement (i.e., the center position of the window), and ξ=(ξ,η) represents the rotated frequency coordinates. This transform effectively combines the rotational property of FRFT with the localization capability of the Gabor window, enabling the robust extraction of features such as oblique textures and image edges. The fractional Gabor window function $g_{\alpha }\left(x,y\right)$ combines a Gaussian envelope with fractional-order phase modulation and is expressed as

(10) $$g_{\alpha }\left(x,y\right)=\exp\left(-\frac{x_{\theta }^{2}+y_{\theta }^{2}}{2\sigma^{2}}\right)\cdot \exp\left[j\pi \left(x_{\theta }^{2}+y_{\theta }^{2}\right)\tan\left(\frac{\alpha }{2}\right)\right]$$

The first term provides the Gaussian envelope-controlling window scale, while the second introduces fractional-order phase modulation determined by the FRFT rotation angle α. This enables orientation-adaptive time–frequency resolution, making the window effective for signals with strong directionality or complex frequency patterns.

While lighter representations like Short-Term Fourier Transform (STFT) or standard Wavelets are effective for stationary texture analysis, they often lack flexibility in capturing the non-stationary and direction-variant characteristics of clothing textures under complex pose deformations. Fabric wrinkles and patterns (e.g., stripes) often exhibit varying orientations that do not align with fixed coordinate axes. The rationale for employing the fractional Gabor transform lies in its unique ability to perform time–frequency analysis in a rotated plane determined by the fractional order α. This provides an additional degree of freedom to optimally align the analysis window with the varying principal directions of the texture, offering a more compact and sparse representation for non-rigidly deformed patterns than isotropic filters or standard wavelets.

### 3.2. Multi-Level Fractional Gabor Frequency Network

**Figure 3:**
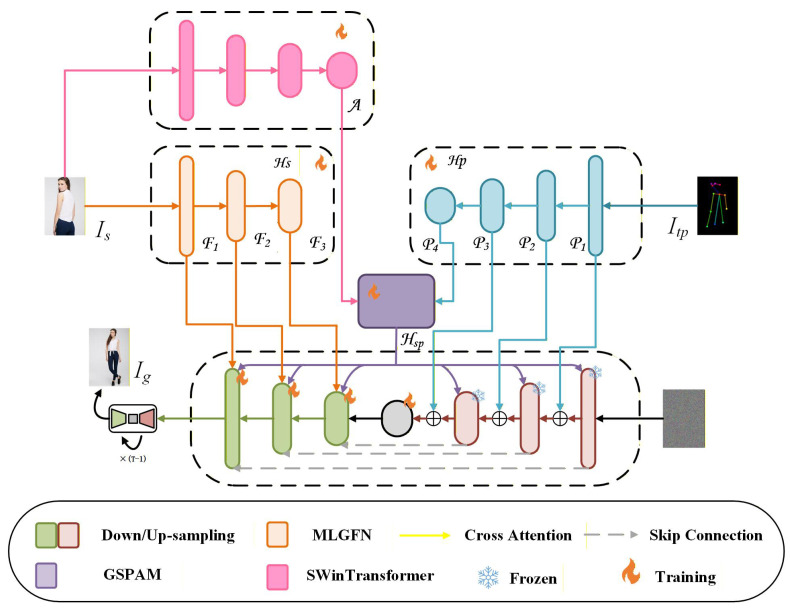
Overview of the proposed FreqPose framework. The source image $I_{s}$ is processed by the Multi-Level Gabor Frequency Network (MLGFN) (orange block) to extract frequency-aware texture features $F_{1-3}$. These features, along with the Pose features $P_{1-4}$ from the Pose Encoder (blue block), are fused via the Global Semantic–Pose Alignment Module (GSPAM) (purple block) before being injected into the denoising UNet. The ’Fire’ icon denotes trainable modules, while the ’Snowflake’ denotes frozen pre-trained weights.

In PGPIS, accurately extracting high-frequency details such as wrinkles, skin texture, and hair is essential for realism and consistency. Existing pre-trained encoders (e.g., VGG and CLIP) often lose fine textures and lack component-level semantic understanding. To address these limitations, we introduce a feature generation framework based on fractional-order Gabor filtering and frequency-domain attention (Figure 3). Specifically, *A* represents the final-layer semantic features extracted via the Swin Transformer, while $\left\{P_{1},P_{2},P_{3},P_{4}\right\}$ denote multi-scale pose features extracted by lightweight ResNet blocks [19]. Furthermore, $\left\{F_{1},F_{2},F_{3},F_{4}\right\}$ correspond to the texture features extracted by our proposed MLGFN. Within the MLGFN, fractional Gabor kernels capture direction- and scale-sensitive texture responses, and a complex-domain attention mechanism aligns global phase information, thereby enabling the accurate preservation of fine details—such as clothing, hair, and edges—even under large pose changes. Finally, $H_{sp}$ denotes the aligned features’ output by our proposed GSPAM after aligning the semantic features with pose features.

We apply transformations to the input at different angles. For each direction θ, the rotated coordinates are defined as

(11) $$\left\{\begin{array}{cc} x_{\theta } & =x\cos\theta +y\sin\theta \\ y_{\theta } & =-x\sin\theta +y\cos\theta \end{array}\right.$$

The angles θ=[0,45,90,135] are used to capture the spatial relationships of textures through four different directions. Based on Euler’s formula, we extend the Gabor window function in Equation (10) to the following form for each angle θ and each scale *s*:

(12) $$\begin{array}{cc} g(x,y;\theta ,\sigma ,\alpha ) & =\exp\left(-{\left(x_{\theta }^{2}+{\left(\frac{y_{\theta }}{s}\right)}^{2}\right)}^{\alpha }\right)\\ \; & \;\cdot \left[\cos\left(\frac{2\pi x_{\theta }}{s}\right)+i\sin\left(\frac{2\pi x_{\theta }}{s}\right)\right] \end{array}$$

The scale s=[1,2,3,4] is used to capture the spatial relationships of textures through four different scales. Although the fractional Gabor transform is defined in the continuous domain, our implementation operates in the discrete space compatible with Convolutional Neural Networks. We discretize the continuous kernel $g_{\alpha }\left(x,y\right)$ by sampling it on a fixed grid of size 3×3. This yields the real and imaginary parts of the Gabor kernel:

(13) $$\left\{\begin{array}{c} g_{\theta ,s}^{R}\left(x,y\right)=G_{\alpha ,\theta ,s}\left(x,y\right)\cos(2\pi \frac{x_{\theta }}{s})\\ g_{\theta ,s}^{I}\left(x,y\right)=G_{\alpha ,\theta ,s}\left(x,y\right)\sin(2\pi \frac{x_{\theta }}{s}) \end{array}\right.$$

where $G_{\alpha ,\theta ,s}\left(x,y\right)=\exp\left(-{\left(x_{\theta }^{2}+{\left(\frac{y_{\theta }}{s}\right)}^{2}\right)}^{\alpha }\right)$. Then, we apply $l_{2}$ normalization:

(14) $$\left\{\begin{array}{c} g_{\theta ,s}^{R}=\frac{g_{\theta ,s}^{R}}{∥g_{\theta ,s}^{R}{∥}_{2}+\epsilon }\\ g_{\theta ,s}^{I}=\frac{g_{\theta ,s}^{I}}{∥g_{\theta ,s}^{I}{∥}_{2}+\epsilon } \end{array}\right.$$

Here, α is the fractional-order parameter, θ and *s* represent the direction and scale, respectively, and ϵ is a stability constant. Compared to the traditional Gabor kernel, fractional-order control allows for the flexible adjustment of the frequency band shape, enhancing the filter’s ability to respond to both fine-grained and coarse-grained textures. This is particularly important for regions such as fabric folds and hair, enabling the model to capture continuous texture patterns across scales, rather than solely focusing on local strong textures.

Based on the above kernel, complex convolution is performed on the input feature *X*.

(15) $$R_{\theta ,s}=\left(X*{\tilde{W}}_{\theta ,s}^{R}\right)+i\left(X*{\tilde{W}}_{\theta ,s}^{I}\right)$$

Here, ∗ denotes the convolution operation, followed by aggregation along the direction and scale dimensions:

(16) $$R=\sum_{\theta \in \Theta }\sum_{s\in S}R_{\theta ,s}$$

where ∑ denotes the summation operator in the equation. This approach not only extracts the magnitude features of the texture but, more importantly, preserves the phase information, thereby capturing the directionality, continuity, and regularity of the texture. This means that when the pose of the person undergoes deformation, local structures such as striped fabrics, hair direction, and clothing folds can still be well preserved, alleviating misalignment and blurring issues.

We further apply GaborFPU to split the input channels into four groups for parallel filtering, followed by fusion through 1×1 convolution and residual mapping:

(17) $$F_{\mathrm{FPU}}=\sigma \left(LN\left(Conv_{1\times 1}\left(\left[Y^{\left(1\right)}⊕Y^{\left(2\right)}⊕Y^{\left(3\right)}⊕Y^{\left(4\right)}\right]\right)\right)+SC\left(X\right)\right)$$

**Figure 4:**
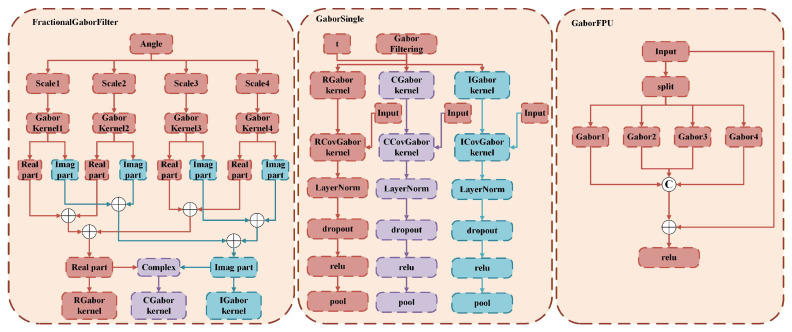
Architectural diagrams of the FractionalGaborFilter, GaborSingle, and GaborFPU modules.

Here, σ represents the ReLU activation, SC(X) denotes the shortcut mapping and ⊕ stands for concatenation. Because each branch processes a distinct orientation and scale, the network learns complementary directional responses; the residual path guarantees that the original feature map is preserved, preventing over-filtering. Through parallel multi-directional filtering and residual design, GaborFPU enhances the texture direction diversity while maintaining overall appearance stability (as shown in Figure 4), providing a structurally richer foundation for the subsequent attention mechanism.

**Figure 5:**
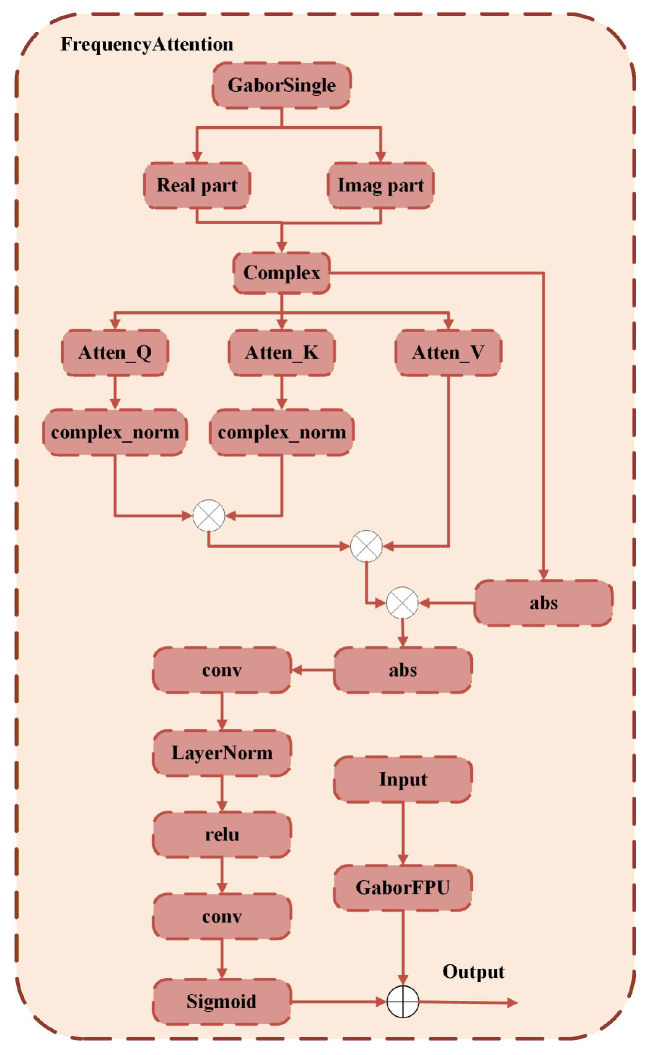
Framework of the FrequencyAttention module.

In global frequency domain modeling, we use FrequencyAttention (as shown in Figure 5) to capture texture phase dependencies across different locations. Let the complex output of GaborSingle be *Z*. We first perform dimensionality reduction on its real and imaginary parts using 1×1 convolution and apply complex normalization to obtain

(18) $$\left\{\begin{array}{c} F=Conv_{R}\left(RZ\right)+iConv_{I}\left(IZ\right)\\ \tilde{F}=\frac{F}{\sum_{c}{\left|F_{c}\right|}^{2}+\epsilon } \end{array}\right.$$

This normalization mitigates the issue of amplitude imbalance across different directional and scale branches, resulting in more stable attention aggregation. Subsequently, we rearrange $\tilde{F}$ into a multi-head format, where N=HW, *h* is the number of heads, and $d=\frac{C^{\prime }}{h}$ is the dimension per head.

(19) $$Q=K=V=\tilde{F}\in C^{B\times h\times N\times d},\;N=H\cdot W,\;d=\frac{C^{\prime }}{h}$$

To perform attention computation in the complex space, we calculate the scaled dot product attention for the real and imaginary parts separately:

(20) $$\left\{\begin{array}{c} A^{R}=\frac{Q^{R}{\left(K^{R}\right)}^{⊤}+Q^{I}{\left(K^{I}\right)}^{⊤}}{\sqrt{dT}}\\ A^{I}=\frac{Q^{I}{\left(K^{R}\right)}^{⊤}-Q^{R}{\left(K^{I}\right)}^{⊤}}{\sqrt{dT}} \end{array}\right.$$

Here, *T* is the temperature coefficient. We apply softmax separately to the real and imaginary attention scores, which empirically yields more stable gradients than a joint complex softmax. After applying softmax to $A^{R}$ and $A^{I}$, the weighted matrices are obtained, with the corresponding real and imaginary parts of the output being

(21) $$\left\{\begin{array}{c} P^{R}=\mathrm{softmax}\left(A^{R}\right)\\ P^{I}=\mathrm{softmax}\left(A^{I}\right) \end{array}\right.$$

Finally, the complex output is obtained as $O=O^{R}+iO^{I}$:

(22) $$\left\{\begin{array}{c} O^{R}=P^{R}V^{R}-P^{I}V^{I}\\ O^{I}=P^{R}V^{I}+P^{I}V^{R} \end{array}\right.$$

This separate computation of the real and imaginary components is more stable than directly applying complex multiplication, while clearly distinguishing in-phase correlations (the real part) from quadrature-phase correlations (the imaginary part). This enables the model to explicitly capture phase alignment relationships within textures, which provides a clear advantage in maintaining texture continuity under large pose variations.

The magnitude is computed as $\left|O\right|=\sqrt{{\left(O^{R}\right)}^{2}+{\left(O^{I}\right)}^{2}}$. Finally, we take the complex magnitude of |O| and fuse it with the channel attention result $W_{c}$ of $|\tilde{F}|$. The fused output is then formed through 1×1 convolution and normalization to produce the frequency attention output:

(23) $$F_{\mathrm{FA}}=LN(Conv_{1\times 1}([|O|,W_{c}⊙|\tilde{F}\left|])\right)+X$$

where ⊙ denotes the Hadamard product, and |·| represents the modulus operator. Channel attention suppresses the irrelevant frequency bands and highlights the texture regions that are related to person identity features, such as fabric patterns, lace, logos, and decorative designs, making the output more focused and expressive. Finally, we fuse $F_{\mathrm{FA}}$ with $F_{\mathrm{FPU}}$:

(24) $$F=LN(F_{\mathrm{FPU}}+F_{\mathrm{FA}})$$

The method achieves dual texture modeling at both the local and global levels. Fractional-order Gabor filtering provides direction-controllable and scale-adaptive complex texture responses at the local level, while FrequencyAttention establishes phase consistency and long-range texture dependencies at the global level. By applying normalization and channel attention, the model’s ability to focus on key high-frequency features is enhanced. The combination of both allows the network to simultaneously preserve the fine structure of local textures and the consistency of global appearance when handling complex pose transformations, significantly improving the clarity and identity fidelity of the generated results.

### 3.3. Global Semantic–Pose Alignment Module

In pose-guided portrait generation, achieving deep alignment between pose structures and semantic features remains challenging. Existing methods often use simple feature concatenation or linear fusion, which fails to preserve spatial correspondences in the deep semantic space, leading to local misalignment and texture drift under large pose variations.

**Figure 6:**
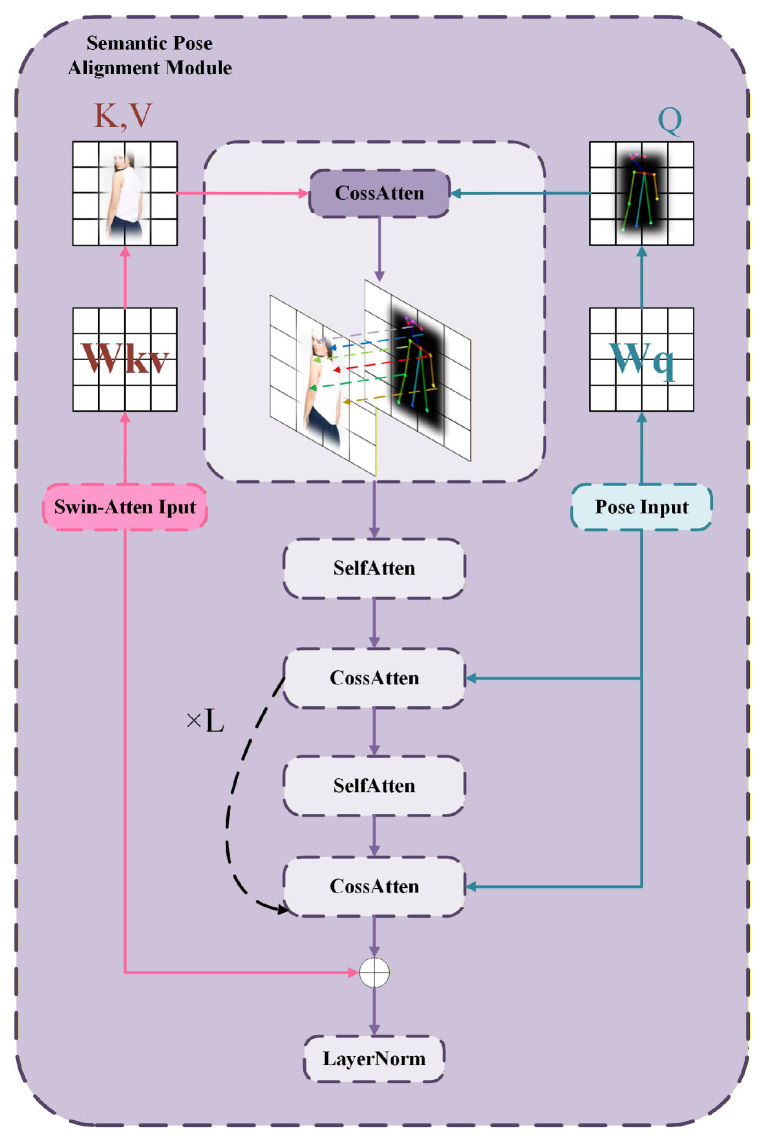
Framework of the Global Semantic–Pose Alignment module.

To effectively align the appearance features of the source image with the target pose, we propose a global semantic–pose alignment module (as shown in Figure 6). This module employs a multi-depth alignment architecture to align the high-level semantic features of the source image with the global spatial guidance information from the target pose map. The aligned features are then injected into the UNet backbone network for pose-guided image synthesis.

First, the pose features undergo 2D average pooling and a 1×1 convolution projection to reduce spatial redundancy and align the channel dimensions, resulting in the deepest layer of pose features:

(25) $$\tilde{P}=Conv_{1\times 1}\left(AvgPool\left(P\right)\right)$$

Then, it is flattened into a sequence of length $n_{q}=H_{q}W_{q}$, followed by layer normalization to obtain the initial query representation:

(26) $$Z_{0}=LN\left(Flatten\left(\tilde{P}\right)\right)$$

Here, LN(.) denotes the layer normalization operation, and latten(.) represents the flattening operation. To retain the spatial relationship corresponding to the query, learnable position embeddings are introduced and added to the query features during subsequent attention computation. This design explicitly preserves topological structural information in pose queries, ensuring that the model does not lose spatial priors of the pose during the global mapping process, thereby enhancing its ability to locate key pose regions.

The deep semantic features of appearance from the Swin Transformer are represented as $A\in R^{B\times n_{k}\times d_{e}}$. To strengthen spatial awareness, key-value position embeddings $K,V\in R^{1\times n_{k}\times d_{e}}$ are added to obtain $\tilde{A}$. This operation allows the appearance features to provide clear spatial anchors in cross-attention, alleviating the misalignment issues caused by significant pose changes.

During the cross-attention phase, let $H_{l}$ be the query at the *l*-th layer. The query, key, and value are first computed through a linear transformation, as follows:

(27) $$\left\{\begin{array}{c} Q_{l}=\left(H_{l}+Q\right)W_{q}\\ K=\tilde{A}W_{k}\\ V=\tilde{A}W_{v} \end{array}\right.$$

Here, $W_{q}\in R^{d\times d}$ and $W_{k}$, $W_{v}\in R^{d_{e}\times d}$ are the projection matrices. Let the number of attention heads be *h*, with the dimension of each head $d_{h}=d/h$. Then, the output of the cross-attention is

(28) $$Attn_{cross}=softmax\left(\frac{QK^{⊤}}{\sqrt{d_{h}}}\right)V$$

The enhanced query representation is then obtained through a residual connection:

(29) $${\tilde{H}}_{l}=H_{l}+Attn_{cross}$$

With this design, pose queries can establish precise correspondences with appearance features on a global scale, achieving structural-level cross-domain alignment. This cross-attention-based global alignment method is more effective than simple feature concatenation, as it fully utilizes the source image information and exhibits stronger robustness to large-scale pose variations. To further enhance the internal consistency of the aligned pose, the model applies self-attention to the query features after the cross-attention stage. Specifically, the query features are obtained through a linear transformation:

(30) $$\left\{\begin{array}{c} {\tilde{Q}}_{l}=\left({\tilde{H}}_{l}+Q\right)U_{q}\\ {\tilde{K}}_{l}=\left({\tilde{H}}_{l}+Q\right)U_{k}\\ {\tilde{V}}_{l}=\left({\tilde{H}}_{l}+Q\right)U_{v} \end{array}\right.$$

Compute self-attention, as follows:

(31) $$Attn_{self}=softmax\left(\frac{{\tilde{Q}}_{l}{\tilde{K}}_{l}^{⊤}}{\sqrt{d_{h}}}\right){\tilde{V}}_{l}$$

Then, update through residual connections and a feedforward network:

(32) $$\left\{\begin{array}{c} H_{l+1}^{\prime }={\tilde{H}}_{l}+Attn_{self}\\ H_{l+1}=H_{l+1}^{\prime }+\mathrm{FFN}\left(LN\left(H_{l+1}^{\prime }\right)\right) \end{array}\right.$$

Here, FFN denotes the feedforward network. The introduction of self-attention establishes rich semantic relationships within the pose query, ensuring that local regions remain highly coordinated and continuous after alignment, thus avoiding issues such as feature fragmentation and local drift. During the output stage, to further enhance the preservation of appearance information, a residual alignment mechanism at the encoding end is introduced, where $\tilde{A}$ is passed through a linear transformation,

(33) $$A_{proj}=\tilde{A}W_{E},$$

and aligns the sequence length to $n_{q}$ through adaptive average pooling:

(34) $$A_{res}=Pool_{n_{k}}\left(A_{proj}\right).$$

Finally, we obtain

(35) $$H_{sp}=LN\left(H_{L}+{\tilde{A}}_{res}\right).$$

where *L* denotes the output of the last Transformer block. The residual fusion between the high-level features of the Swin Transformer and the pose queries provides a direct appearance feedback pathway beyond attention alignment, which enhances the preservation of critical appearance information and improves both the gradient stability and training convergence. The entire alignment process is composed of four sequential stages including pose querying, global cross-attention, local self-attention, and residual fusion, through which structural and appearance information are efficiently coupled. The cross-attention mechanism ensures global geometric and appearance correspondence, the self-attention mechanism refines local semantic consistency, and the residual pathway further stabilizes information propagation. This design effectively reduces structural misalignment and texture drift under complex pose variations, achieving higher alignment accuracy and better detail fidelity.

### 3.4. Optimization Objectives and Sampling Strategies

In this work, the initial latent state of the real image is defined as $z_{0}=\varepsilon \left(I_{t}\right)$. Therefore, the mean squared error loss in Equation (2) can be re-expressed as

(36) $$L_{mse}=E_{z_{0},I_{s},I_{sp},\varepsilon ,t}\left[{∥\varepsilon -\varepsilon_{\theta }\left(z_{t},t,I_{s},I_{tp}\right)∥}_{2}^{2}\right]$$

To facilitate the transfer from the source pose to the target pose, as suggested in [20], a source-to-source self-reconstruction task is introduced during the training process. The corresponding reconstruction loss is expressed as

(37) $$L_{rec}=E_{z_{0},I_{s},I_{sp},\varepsilon ,t}\left[{∥\varepsilon -\varepsilon_{\theta }\left(z_{t},t,I_{s},I_{sp}\right)∥}_{2}^{2}\right]$$

Here, $Z_{0}=\varepsilon \left(I_{s}\right)$, and $z_{t}$ represents the latent representation obtained at time step *t* after noise is injected into $z_{0}$. Therefore, the overall objective function can be expressed as

(38) $$L_{overall}=L_{mse}+L_{rec}$$

Therefore, the overall objective function is defined as the sum of the mean squared error loss and the reconstruction loss. As mentioned, once the conditional latent variable diffusion model is trained, the inference process can begin. Specifically, sampling starts from Gaussian noise, i.e., $Z_{T}∼N\left(0,I\right)$, and at each time step t∈[1,T], the denoising network $\varepsilon_{t}$ is applied to reverse Equation (1), thereby obtaining the predicted latent representation ${\tilde{z}}_{0}$. To simultaneously enhance the source image appearance and the guidance effect of the target pose, this study adopts a classifier-free guidance accumulation strategy, which is computed as follows:

(39) $$\begin{array}{cc} \epsilon_{t} & =\epsilon_{\theta }\left(z_{t},t,⌀,⌀\right)\\ \; & \;+w_{pose}\left(\epsilon_{\theta }\left(z_{t},t,⌀,I_{tp}\right)-\epsilon_{\theta }\left(z_{t},t,⌀,⌀\right)\right)\\ \; & \;+w_{app}\left(\epsilon_{\theta }\left(z_{t},t,I_{s},I_{tp}\right)-\epsilon_{\theta }\left(z_{t},t,⌀,I_{tp}\right)\right) \end{array}$$

When the source image $I_{s}$ is unavailable, a learnable vector is used as the conditional embedding. During training, with probability $\eta_{\%}$, both $I_{s}$ and $I_{tp}$ are randomly dropped to achieve regularized learning. If the target pose $I_{tp}$ is missing, the output of the pose adapter is set to zero. To accelerate the sampling process, this study adopts the DDIM scheduler [21] with 50 sampling steps, consistent with [8,11]. Finally, the generated image is obtained through the VAE decoder, i.e., $I_{g}=D\left({\tilde{z}}_{0}\right)$. Additionally, the distribution strategy of the time steps follows a cubic function form:

(40) $$t=\left(1-{\left(\frac{t}{T}\right)}^{3}\right)\times T,\;t\in Uniform\left(1,T\right)$$

Overall, this strategy increases the probability of the time steps falling in the early sampling stages, thereby enhancing the model’s guidance capability, promoting faster convergence, and effectively reducing training time. In summary, the proposed MultiLevelGaborNet is outlined in Algorithm 1.
**Algorithm 1:** Multi-Level Gabor Frequency Network    **Input:** Source image $I_{s}\in R^{3\times H\times W}$; fractional order α; angle set          Θ={0°,45°,90°,135°}; scale set S={1,2,3,4}.    **Output:** Multi-level frequency-aware features $\left[F_{1},F_{2},F_{3}\right]$.   **1** **Stage 1: Patch Embedding and Local Frequency Encoding**   **2** $X_{1}\leftarrow \mathrm{PatchEmbed}\left(I_{s}\right)$;              // Extract low-level patches   **3** $Y_{1}\leftarrow \mathrm{GaborFrequencyNet}\left(X_{1}\right)$   **4**    Apply fractional Gabor filters $G_{\alpha }\left(\theta ,s\right)$ to capture orientation–scale textures;   **5**    Fuse complex-domain features via Frequency Attention (FA):   **6** $Y_{1}\leftarrow \alpha_{1}\cdot \mathrm{FA}\left(G_{\alpha }\left(X_{1}\right)\right)+\left(1-\alpha_{1}\right)\cdot G_{\alpha }\left(X_{1}\right)$   **7** **Stage 2: Mid-Level Aggregation**   **8** $X_{2}\leftarrow \mathrm{PatchMerging}\left(Y_{1}\right)$;          // Downsample and double channels    **9** $Y_{2}\leftarrow \mathrm{GaborFrequencyNet}\left(X_{2}\right)$ **10** **Stage 3: High-Level Fusion** **11** $X_{3}\leftarrow \mathrm{PatchMerging}\left(Y_{2}\right)$ **12** $Y_{3}\leftarrow \mathrm{GaborFrequencyNet}\left(X_{3}\right)$ **13** **Feature Reshaping** **14** $F_{1}\leftarrow \mathrm{Reshape}\left(Y_{1}\right)\in R^{4096\times 128}$; $F_{2}\leftarrow \mathrm{Reshape}\left(Y_{2}\right)\in R^{1024\times 256}$; $\;F_{3}\leftarrow \mathrm{Reshape}\left(Y_{3}\right)\in R^{256\times 512}$ **15** **return** $\left[F_{1},F_{2},F_{3}\right]$

## 4. Experimental Results and Discussion

### 4.1. Experimental Procedure

All the experiments follow the In-Shop Clothes Retrieval benchmark on DeepFashion [22] with the protocol of [11,21], using resolutions of 256 × 176 and 512 × 352. The dataset contains 52,712 images, split into 101,966 training and 8570 testing pairs following [23]. Market-1501 [24], with lower resolution and more complex backgrounds, and uses 263,632 training and 12,000 testing pairs under the same split strategy [23].

Quantitative evaluation employs FID [25], LPIPS [26], SSIM [27], and PSNR, where FID and LPIPS measure deep feature realism and perceptual similarity, while SSIM and PSNR assess pixel-level reconstruction. Following [11], user studies adopt the Jab indices [8,28,29] to compute the proportion of images judged best, and R2G/G2R metrics [23,30] quantify real–generated indistinguishability.

### 4.2. Implementation Details

All the experiments were conducted on four NVIDIA RTX 4090 GPUs with 24 GB of memory, using Python 3.10, PyTorch 2.0 [31], and the HuggingFace Diffusers framework [32]. The core generative model was based on Stable Diffusion v1.5 [2]. The input image resolution was standardized to 256 × 256. Table 1 summarizes the default settings and the number of trainable parameters for each component.

During training, the Adam optimizer [33] was employed for a total of 160 epochs, with an initial learning rate of $5\times 10^{-5}$, scaled according to the overall batch size. A linear warm-up strategy was applied for the first 1000 steps, followed by a learning rate decay by a factor of 0.1 at the 80th epoch. During inference, to enhance structural consistency and pose control, we adopted a classifier-free guidance mechanism, setting both the pose prompt weight $W_{pose}$ and and the appearance prompt weight $W_{id}$ to 2.0. Moreover, to improve model robustness and generalization, conditional perturbation training was applied by randomly dropping the conditional inputs $I_{s}$ and $I_{tp}$ with a probability of η=20% during training.

### 4.3. Comparative Experiments

#### 4.3.1. Quantitative Results

**DeepFashion**: On the DeepFashion dataset, the proposed method was compared with existing PGPIS baselines, including PATN [23], ADGAN [20], GFLA [34], PISE [35], SPGNet [28], DPTN [36], NTED [37], CASD [29], PIDM [8], PoCoLD [11], and CFLD [10]. To ensure fair comparison, the results of these methods were obtained using the officially released source codes and pre-trained checkpoints provided by the respective authors. Among them, PATN, ADGAN, XingGAN, and GFLA are representative GAN-based approaches, whereas PoCoLD, PIDM, and CFLD are diffusion model-based PGPIS methods. The evaluation was conducted on test images with resolutions of 256 × 176 and 512 × 352.

Table 2 and Table 3 report the quantitative evaluation results of our method on the DeepFashion dataset at two resolutions, 256 × 176 and 512 × 352. The comparison metrics include FID, LPIPS, SSIM, and PSIM. Overall, our method consistently outperforms existing methods across all four metrics at both resolutions, demonstrating comprehensive performance advantages.

It is noteworthy that even compared to diffusion model-based methods (e.g., PIDM and PoCoLd), our approach still shows superior performance. While these models have made improvements in image realism, their limited conditional control mechanisms prevent them from fully integrating the global semantic information of the source image with the structural features of the target pose, leading to only marginal improvements in perceptual similarity metrics. In contrast, our MLGFN and GSPAM significantly enhance the accuracy of source image detail and structure restoration, while maintaining high image realism.

In the overall architecture, MLGFN achieves the multi-scale modeling of high-frequency features, such as clothing textures and hand details, through fractional-order Gabor filters and frequency-domain attention mechanisms, improving the detail fidelity and structural stability of the generated images. GSPAM establishes a global mapping between pose and appearance features at the semantic level, using cross-modal feature interaction and attention alignment mechanisms to precisely fuse the appearance information of the person with the target pose, alleviating the appearance distortion issues under large pose variations. The synergy of these two components ensures that the model maintains geometric consistency and structural integrity in regions such as joints and hands, even in complex scenes, and significantly improves structural consistency metrics (e.g., SSIM). This demonstrates exceptional cross-pose generalization and high-fidelity generation performance.

#### 4.3.2. Market-1501

On the Market-1501 dataset at 128 × 64 resolution (Table 4), our method was benchmarked against state-of-the-art models including GFLA, XingGAN, SPGNet, PIDM, CFLD, and DPTN. Across all four quantitative metrics, our approach consistently achieves the best performance, with particularly large gains in FID and LPIPS, indicating superior realism, distribution alignment, and perceptual detail preservation. The higher SSIM and PSNR scores further confirm stronger structural consistency and pixel-level reconstruction accuracy. These results collectively demonstrate the effectiveness of the proposed multi-level fractional-order Gabor frequency-aware network and semantic–pose alignment module in restoring fine details and achieving robust cross-pose generalization for low-resolution pedestrian image generation in complex backgrounds.

#### 4.3.3. Qualitative Results

**Figure 7:**
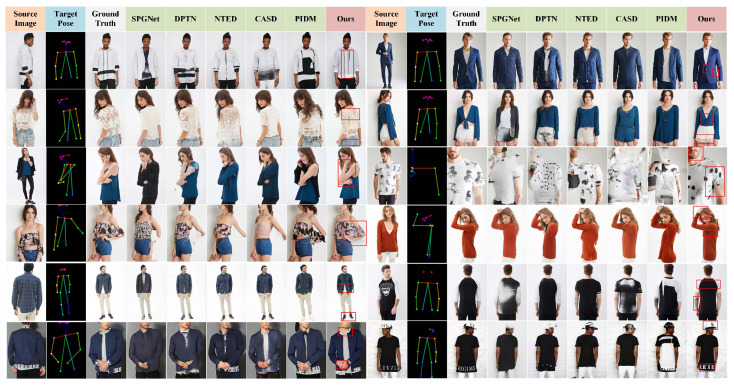
Visual comparison between our method and state-of-the-art methods.

Figure 7 presents a visual comparison of the results obtained by our method and other state-of-the-art methods, including SPGNet, DPTN, NTED, CASD, GFLA, XingGAN, and PIDM.

**Figure 8:**
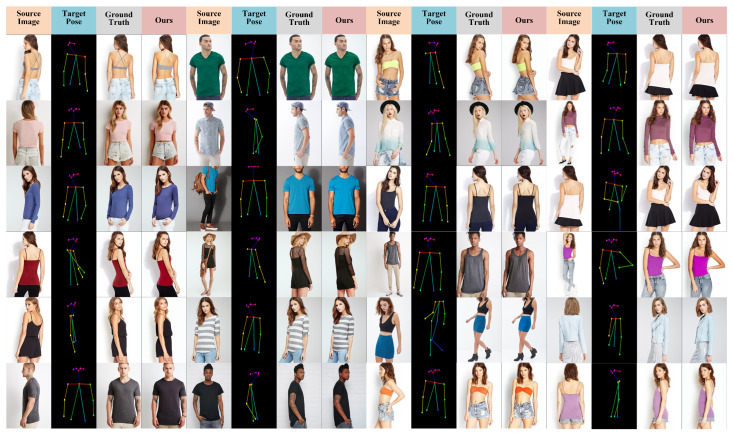
Generated results of our method.

As shown in Figure 7 (rows 1–2), our method demonstrates superior structural preservation and high-frequency texture restoration on source images with rich details, effectively suppressing artifacts such as fractures and blurriness. This benefit mainly comes from the MLGFN, whose adaptive fractional-order frequency-domain filtering and multi-branch frequency-aware modeling enable the precise capture of non-stationary textures. In structure-sensitive regions—including hands, clothing edges, and patterned textures—as shown in Figure 7 (rows 3–4), traditional GAN- and diffusion-based baselines often suffer from disconnections, blurring, or misalignment, whereas the proposed GSPAM establishes a global semantic mapping between pose and appearance through cross-modal attention and Swin Transformer-based feature interactions, achieving accurate appearance alignment and improved structural coherence. Even under large pose variations or occlusions, as shown in Figure 7 (rows 5–6), combining MLGFN’s multi-level frequency-aware features with GSPAM’s semantic–pose alignment representations forms a unified conditional modulation for the Stable Diffusion denoising process, ensuring consistent structure and realistic texture reconstruction in challenging scenarios. Additional qualitative results are provided in Figure 8.

To comprehensively evaluate the computational cost and scalability of our proposed framework, we conducted a comparative analysis of model parameters, Floating Point Operations (FLOPs), and inference latency against the state-of-the-art diffusion baseline PIDM, the pose-constrained latent diffusion model PoCoLD, and the lightweight method CFLD. The experiments were performed on a single NVIDIA RTX 4090 GPU at two standard resolutions: 256×176 and 512×352. Quantitative Comparison: As presented in Table 5, our FreqPose model contains 277.6 M parameters. This makes it significantly more parameter efficient than both PIDM (688.0 M) and PoCoLD (395.9 M), while being comparable to the lightweight CFLD (248.2 M). At low resolution (256×176), our method requires 112.4 G FLOPs and 0.52 s per image. This performance is highly competitive with PoCoLD (100.0 G; 0.50 s) and CFLD (89.5 G; 0.45 s). The marginal overhead (∼15% vs. CFLD) is primarily attributed to the multi-level fractional Gabor calculations in the MLGFN module, yet it remains well within the range for real-time applications. At high resolution (512×352), the computational advantage over heavy diffusion models becomes more pronounced. PIDM requires 4.50 s per image, making it impractical for efficient deployment, whereas FreqPose achieves high-fidelity generation in 1.87 s, maintaining a reasonable inference speed comparable to lighter methods. Trade-off Analysis: While FreqPose introduces a slight computational cost over the lightest baselines, this trade-off is fully justified by the substantial performance gains. For instance, although PoCoLD achieves a similar inference speed to our method, its texture fidelity is limited (SSIM 0.731 in Table 2). In contrast, FreqPose achieves superior quality (SSIM 0.769), outperforming PoCoLD and CFLD by a clear margin. The proposed framework thus strikes an optimal balance, delivering state-of-the-art generation quality with compact parameter size and inference speeds suitable for practical deployment.

### 4.4. Ablation Experiments

To validate the superiority of the proposed MLGFN and GSPAM in texture feature extraction and semantic alignment, we conducted a series of systematic ablation experiments across multiple feature encoding baselines. As shown in Table 6, we configured five comparison setups. Baseline B1 uses VAE to directly encode the source image, B2 employs the CLIP image encoder to extract features from the source image, B3 follows mainstream diffusion approaches [8,11], utilizing multi-scale features extracted by general pre-trained models such as Swin Transformer as conditional embeddings, B4 represents the proposed method, which utilizes a multi-level fractional-order Gabor frequency-aware network as the core for texture feature extraction, with its output serving as the bias term for the query in the UNet cross-attention mechanism to achieve fine-grained control of texture details, and B5 employs the traditional CLIP image encoder for texture extraction from the source image, while incorporating our proposed GSPAM for semantic alignment. Finally, B6 represents the complete model proposed in this paper.

**Figure 9:**
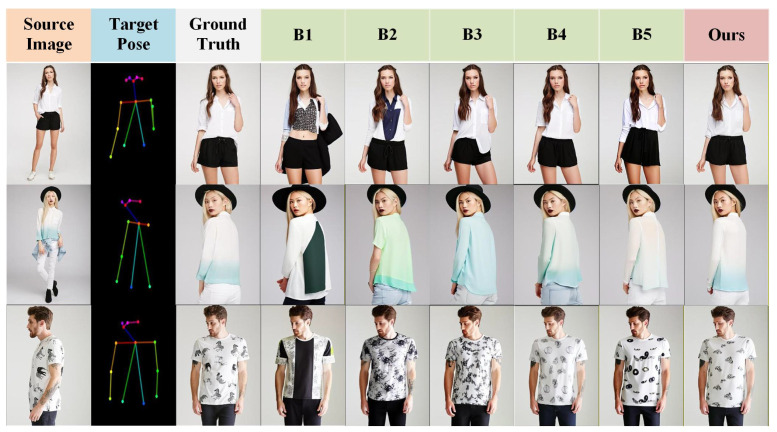
Visualization of ablation experiment.

The experimental results (visualized in Figure 9) indicate that configurations based on VAE and CLIP (B1 and B2) perform the weakest in terms of texture restoration, with even simple fabric textures failing to be fully preserved. This highlights the clear modal differences between general-purpose visual models and diffusion generation tasks. While the Swin Transformer architecture used in B3 has advantages in semantic understanding, it still has inherent limitations in high-frequency texture modeling. In contrast, the frequency-aware network (B4) proposed in this paper demonstrates significant improvements in reconstruction metrics like SSIM and LPIPS, fully proving its exceptional ability in high-frequency detail capture and restoration. Its performance not only surpasses all baselines but also underscores the irreplaceable advantage of a frequency-aware module specifically designed for texture characteristics, compared to general pre-trained encoders, in generation tasks. B5 further builds upon this by introducing the Global Semantic–Pose Alignment Module, explicitly establishing cross-modal correspondences between pose and appearance in the high-level semantic space, achieving the simultaneous optimization of structural consistency and global coherence in the generated results, thereby further enhancing the overall perceptual quality of cross-pose person image synthesis.

**Fine-grained Ablation Study.** To further investigate the effectiveness of the internal designs within MLGFN and GSPAM, we conducted a component-wise ablation study on the DeepFashion dataset. The results are reported in Table 7.

(1) Effectiveness of MLGFN Components: We analyzed two variants to validate the texture extraction module:

- w/o Fractional Order (α=1): We fixed the fractional order α to 1, effectively degrading the fractional Gabor filters to standard Gabor filters. As shown in Table 7, SSIM drops from 0.769 to 0.748. This decline confirms that the rotational flexibility provided by the fractional order is crucial for capturing non-stationary textures like fabric wrinkles, which standard isotropic filters fail to model effectively.
- w/o Frequency Attention: Removing the frequency-domain attention mechanism leads to a performance drop (LPIPS increases to 0.151). This indicates that the global phase alignment captured by the attention module is essential for maintaining texture continuity and reducing artifacts.

(2) Effectiveness of GSPAM Components: We evaluated the contribution of the cross-modal alignment strategy:

- w/o Cross-Attention: Replacing the cross-attention mechanism with simple feature concatenation results in a significant degradation (SSIM drops to 0.741). This validates our hypothesis that direct concatenation cannot establish precise spatial correspondence between the source appearance and target pose, leading to structural misalignment.
- w/o Self-Attention: Removing the subsequent self-attention layer also hurts performance (SSIM 0.758), suggesting that refining local semantic consistency within the pose queries is beneficial for generating coherent body structures.

### 4.5. User Study

**Figure 10:**
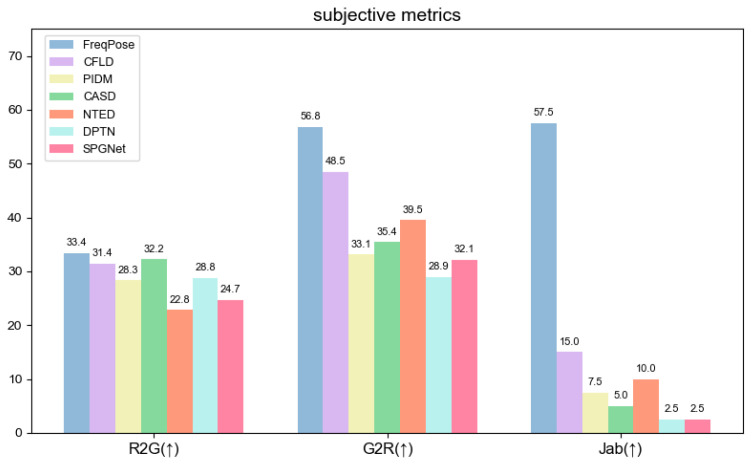
Our method is compared with the excellent methods in terms of subjective metrics.

To evaluate the perceptual realism of the generated images and the superiority of our method, we conducted two user studies following the PIDM [8] standard, with a total of 120 participants:

(1) Realism Test (R2G/G2R):

The participants were asked to determine whether the images were real photographs or model generated. The results, as shown in Figure 10, reveal that our method achieved a G2R score exceeding 55%, significantly outperforming the comparison methods. The generated images were perceived as more realistic, with the textures, structures, and lighting details more closely resembling real images. The lower R2G score indicates that participants were still able to accurately identify real images, validating that the model improvements stem from objective perceptual optimization. This advantage is attributed to MLGFN’s precise modeling of high-frequency textures and GSPAM’s constraints on structural consistency.

(2) Subjective Preference Test (Jab):

From the results of multiple methods, participants selected the image most similar to the target pose. Our method achieved a Jab score of 57.5%, a 42.5% improvement over the next best method, excelling in texture reconstruction, pose alignment, and semantic consistency. Qualitative feedback highlighted that our method produced natural transitions in joint connections and edge details, demonstrating the synergistic advantage of MLGFN and GSPAM.

In summary, both in terms of perceptual realism (G2R/R2G) and subjective preference (Jab), our method significantly outperforms existing approaches, showcasing the visual credibility and application potential of high-fidelity cross-pose person image generation.

## 5. Conclusions

This study systematically addresses the issues of visual distortion and structural inconsistency in pose-guided person image generation, arising from insufficient texture modeling and inadequate semantic alignment. To tackle these challenges, we propose a diffusion-based generative framework that integrates frequency awareness and semantic alignment. By introducing a MLGFN and a semantic–pose alignment module, our method significantly enhances the model’s ability to perform high-frequency texture modeling and cross-pose semantic mapping. MLGFN employs fractional-order Gabor filtering and complex-domain self-attention to robustly extract clothing textures and edge details in the frequency domain, while the semantic–pose alignment module leverages hierarchical semantic features from the Swin Transformer to achieve precise correspondence between appearance and target pose. The experimental results on the DeepFashion and Market-1501 datasets demonstrate that our method outperforms existing approaches in multiple objective metrics and subjective visual quality, showing clear advantages in complex texture reconstruction and large pose transformation tasks. Future work will explore the integration of explicit 3D human priors and multimodal semantic guidance mechanisms to further enhance the model’s generalization and generation stability.

## Figures and Tables

**Table 1:** The number of trainable parameters for each component.

Component	Default	Trainable Params
MLGN	N=3	28.0 M
GSPAM	R=8	103.3 M
Swin-B	L=4	87.0 M
$H_{up}$	$W_{l}^{K},W_{l}^{V}$	18.4 M
$H_{p}$	Adapter	30.6 M
$H_{n}$	$W^{K},W^{V}$	10.3 M

**Table 2:** Comparison of our method with advanced approaches on the DeepFashion dataset at a resolution of 256 × 176.

Method	Venue	FID ↓	LPIPS ↓	SSIM ↑	PSNR ↑
*Evaluate on 256 × 176 resolution*					
PATN	CVPR19	20.731	0.2531	0.6719	-
ADGAN	CVPR20	14.545	0.2245	0.6738	-
GFLA	CVPR20	9.817	0.1863	0.7062	-
PISE	CVPR21	11.520	0.2251	0.6437	-
SPGNet	CVPR21	16.183	0.2256	0.6959	17.222
DPTN	CVPR22	17.409	0.2087	0.6978	17.811
NTED	CVPR22	8.518	0.1757	0.7148	17.740
CASD	ECCV22	13.137	0.1781	0.7223	17.880
PIDM	CVPR23	6.812	0.2106	0.6631	15.630
PoCoLD	ICCV23	8.069	0.1642	0.7314	-
CFLD	CVPR24	6.804	0.1519	0.7368	18.235
**Ours**	-	**6.741**	**0.1408**	**0.7687**	**18.839**
Ground Truth	-	7.847	0.0000	1.0000	+∞

**Table 3:** Comparison of our method with advanced approaches on the DeepFashion dataset at a resolution of 512 × 352.

Method	Venue	FID ↓	LPIPS ↓	SSIM ↑	PSNR ↑
*Evaluate on 512 × 352 resolution*					
CoCosNet2	CVPR21	13.315	0.2271	0.7226	-
NTED	CVPR22	7.621	0.1998	0.7365	17.385
PoCoLD	ICCV23	8.411	0.1923	0.7433	-
CFLD	CVPR24	7.150	0.1817	0.7488	17.645
**Ours**	-	**7.008**	**0.1672**	**0.7694**	**18.110**

**Table 4:** Comparison of the proposed method with state-of-the-art approaches on the Market-1501 dataset at a resolution of 128 × 64.

Method	Venue	FID ↓	LPIPS ↓	SSIM ↑	PSNR ↑
*Evaluate on 128 × 64 resolution*					
GFLA	CVPR20	19.741	0.2825	0.2808	14.337
XingGAN	ECCV20	22.520	0.3049	0.3044	14.446
SPGNet	CVPR21	23.517	0.2787	0.3139	14.489
DPTN	CVPR22	18.996	0.2712	0.2854	14.521
PIDM	CVPR23	14.451	0.2415	0.3054	-
CFLD	CVPR24	11.972	0.2636	0.2854	15.337
**Ours**	-	** 11.821**	**0.2451**	**0.3263**	**15.349**
Ground Truth	-	4.845	0.0000	1.0000	+∞

**Table 5:** Efficiency and performance comparison with state-of-the-art methods at different resolutions. Metrics are measured on a single NVIDIA RTX 4090. FreqPose achieves the best trade-off, maintaining efficient inference speed at high resolutions while delivering superior texture fidelity.

Method	Params (M)	256×176 Efficiency			512×352 Efficiency			Performance (512×352)	
		FLOPs (G)	Time (s)	Mem (GB)	FLOPs (G)	Time (s)	Mem (GB)	FID↓	SSIM↑
PIDM	688.0	185.3	1.25	8.5	741.2	4.50	14.2	-	-
PoCoLD	395.9	100.0	0.50	6.8	400.0	1.80	10.5	8.41	0.743
CFLD	248.2	89.5	0.45	5.8	358.0	1.62	9.0	7.15	0.748
**FreqPose (Ours)**	**277.6**	**112.4**	**0.52**	**9.2**	**449.6**	**1.87**	**13.5**	**7.01**	**0.769**

**Table 6:** Ablation experiment.

Method	Embedding	LPIPS ↓	SSIM ↑
B1	VAE	0.2021	0.6948
B2	CLIP	0.2093	0.6948
B3	Swin-B	0.1652	0.7121
B4	MLGFN	0.1468	0.7399
B5	GSPAM	0.1493	0.7128
B6 (Ours)	FreqPose	0.1408	0.7687

**Table 7:** Component-wise ablation study of MLGFN and GSPAM on the DeepFashion dataset. The results validate the necessity of each internal design choice.

Module	Variant	LPIPS ↓	SSIM ↑
MLGFN	w/o Fractional Order (α=1)	0.155	0.748
	w/o Frequency Attention	0.151	0.753
GSPAM	w/o Cross-Attention (Concat)	0.162	0.741
	w/o Self-Attention	0.148	0.758
**Full Model**	**Proposed Method**	**0.1408**	**0.7687**

## Data Availability

Publicly available datasets were analyzed in this study. The data can be found at the following sources: DeepFashion (https://mmlab.ie.cuhk.edu.hk/projects/DeepFashion.html, accessed on 27 June 2016) and Market-1501 (https://www.kaggle.com/datasets/pengcw1/market-1501, accessed on 7 December 2015). The project code used to support the findings of this study is available at (https://github.com/bing32475/FrGPose) (accessed on 15 February 2026).
